# 174. Development of a Machine Learning Prediction Model to Select Empirical Antibiotics in Patients with Clinically Suspected Urinary Tract Infection using Urine Culture Data

**DOI:** 10.1093/ofid/ofab466.376

**Published:** 2021-12-04

**Authors:** Chungsoo Kim, Rae Woong Park, Sandy J Rhie

**Affiliations:** 1 Ajou University, Suwon-si, Kyonggi-do, Republic of Korea; 2 Ewha Womans University, Seoul, Seoul-t’ukpyolsi, Republic of Korea

## Abstract

**Background:**

Increasing antimicrobial resistance and the emergence of superbugs are problems globally. Inappropriate empiric antibiotic use would be a reason to cause antibiotic resistance. However, it has been a challenge to prescribe empiric antibiotics as it is difficult to identify the causative organism beforehand. In this study, we aimed to develop a prediction model to estimate the risk of antibiotics resistance using urine culture tests.

**Methods:**

The study population included adult patients who had at least one of the results from a urine culture test and antibiotic susceptibility tests (from ampicillin, ceftriaxone, ciprofloxacin, gentamicin, levofloxacin, nitrofurantoin, tetracycline, trimethoprim/sulfamethoxazole) on admission to Ajou University Medical Center. Outcomes were defined as a resistant or intermediate susceptibility. Candidate predictors were diagnosis, prescription, visit, laboratory, procedures of the study population. We split data to 75:25 for training and test. Lasso logistic regression (LLR), extreme gradient boosting machine (XGB), Random Forest (RF) were used as model algorithms. The models were evaluated by an area under the curve of receiver operator characteristics curve (AUROC), precision-recall curve (AUPRC), and its calibration. All codes are available in https://github.com/ABMI/AbxBetterChoice

**Results:**

Total 33 covariates were selected for final prediction models. The RF showed the highest AUROC in the ceftriaxone and tetracycline models (0.823, 0.626, respectively). The XGB presented the highest AUROC for ciprofloxacin and nitrofurantoin (0.731, 0.706, respectively). The AUROC of RF and the XGB were the same in an ampicillin model (0.633). For gentamicin, levofloxacin, and trimethoprim/sulfamethoxazole, the AUROC of LLR was the highest (0.838, 0.831, 0.615, respectively). Among the models, the AUROC was the highest in the gentamicin model regardless of algorithms. All calibrations of the models were acceptable.

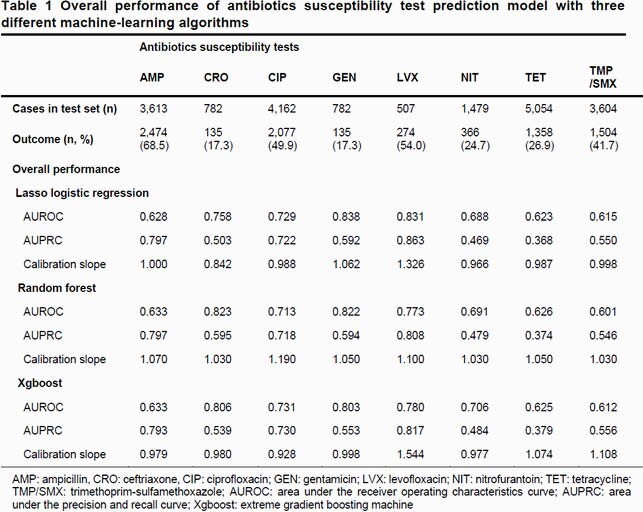

**Conclusion:**

We developed prediction models with competing performances of discrimination and calibration. It would contribute to the proper selection of empiric antibiotics susceptible to those causative pathogens in hospitalized patients with a clinically suspected urinary tract infection.

**Disclosures:**

**All Authors**: No reported disclosures

